# Photodisruption of the Inner Limiting Membrane: Exploring ICG Loaded Nanoparticles as Photosensitizers

**DOI:** 10.3390/pharmaceutics14081716

**Published:** 2022-08-17

**Authors:** Kaat De Clerck, Geraldine Accou, Félix Sauvage, Kevin Braeckmans, Stefaan C. De Smedt, Katrien Remaut, Karen Peynshaert

**Affiliations:** 1 Lab of General Biochemistry and Physical Pharmacy, Faculty of Pharmaceutical Sciences, Ghent University, Ottergemsesteenweg 460, 9000 Ghent, Belgium; 2 Ghent Research Group on Nanomedicines, Ghent University, Ottergemsesteenweg 460, 9000 Ghent, Belgium; 3 Department of Ophthalmology, Ghent University Hospital, 9000 Ghent, Belgium

**Keywords:** retinal drug delivery, inner limiting membrane, photodisruption, vapor nanobubbles, indocyanine green, nanotechnology, pulsed laser

## Abstract

The inner limiting membrane (ILM) represents a major bottleneck hampering efficient drug delivery to the retina after intravitreal injection. To overcome this barrier, we intend to perforate the ILM by use of a light-based approach which relies on the creation of vapor nanobubbles (VNBs) when irradiating photosensitizers with high intensity laser pulses. Upon collapse of these VNBs, mechanical effects can disrupt biological structures. As a photosensitizer, we explore indocyanine green (ICG) loaded nanoparticles (NPs) specifically designed for our application. In light of this, ICG liposomes and PLGA ICG NPs were characterized in terms of physicochemical properties, ICG incorporation and VNB formation. ICG liposomes were found to encapsulate significantly higher amounts of ICG compared to PLGA ICG NPs which is reflected in their VNB creating capacity. Since only ICG liposomes were able to induce VNB generation, this class of NPs was further investigated on retinal explants. Here, application of ICG liposomes followed by laser treatment resulted in subtle disruption effects at the ILM where zones of fully ablated ILM were alternated by intact regions. As the interaction between the ICG liposomes and ILM might be insufficient, active targeting strategies or other NP designs might improve the concept to a further extent.

## 1. Introduction

The global burden of vision impairment and blindness is estimated to afflict the increasingly elderly population on a continuous basis in the coming years with the most prominent pathologies being glaucoma, age-related macular degeneration, diabetic retinopathy along with several inherited disorders [1,2]. All these diseases share the origin of their underlying mechanism at the level of the retina, a key player in visual processing. Although, while worldwide scientific effort continuously uncovers potential therapies such as gene- and stem cell therapy products to treat retinal diseases [3,4], efficient delivery to the posterior segment of the eye is essential for their clinical translation. Following the footsteps of the first FDA approved retinal gene therapy product Luxturna, clinical trials are currently to a large extent monopolized by gene augmentation strategies targeting the outer retina via subretinal injection. However, while highly effective to target photoreceptor cells (PRs) and the retinal pigment epithelium (RPE), new pioneering therapeutic strategies such as neuroprotection [5], optogenetics [6] and reprogramming [7] demand delivery to target cells located in the inner retina. In this regard, intravitreal (IVT) injection is increasing in interest given its low-invasive and easy-to-perform character which is exemplified by its routinely clinical use to deliver antibodies [8]. Despite these attractive features, large therapeutics in the nanosized-range and beyond struggle to reach their target cells after injection into the vitreous. The major culprit challenging retinal delivery after IVT injection is the inner limiting membrane (ILM), a thin membrane located in front of the retina [9,10,11]. While several inventive approaches to overcome this barrier such as subILM injection [12], ILM peeling [13] and enzymatic digestion [10] indeed elicit enhanced drug delivery, the lack of tunability and/or highly invasive nature of these approaches block the road to clinical translation.

Conscious of the need for new perspectives, our group recently explored an innovative light-based approach to disrupt the ILM in a controlled manner. In general, this physical technique is based on pulsed laser irradiation combined with photothermal molecules or nanoparticles (NPs) and is mainly investigated as a tool to enhance intracellular drug delivery, termed photoporation for this purpose [14,15,16]. The procedure is usually initiated by incubation of cells with a photosensitizer followed by application of extremely short laser pulses (<10 ns). Thereby, an ultrafast increase in temperature is induced resulting in evaporation of the surrounding water. Consequently, nanoscopic bubbles, known as vapor nanobubbles (VNBs), are created which expand by consuming their thermal energy. Finally, upon their collapse, high-pressure shock waves are released which can create transient pores in the cell membrane. Seeing that the concept recently broadened its applications to destruct larger biological structures such as biofilms [17] and collagen aggregates in the vitreous (‘eye floaters’) [18,19,20], laser-induced VNB formation also offers an interesting alternative to locally disrupt the main delivery bottleneck after IVT injection, i.e., the ILM.

Based on their intrinsic capacity to efficiently convert absorbed laser light into photothermal phenomena, plasmonic materials—e.g., gold nanoparticles (AuNPs)—are well suited to expertly mediate VNB formation due to their localized surface plasmon resonance (LSPR). However, as toxicity-concerns [21] as well as lack of biodegradability interfere clinical translation, a shift toward the design of photosensitizing materials with biodegradable and biocompatible characteristics is on the rise [22]. For ILM disruption specifically, the FDA approved organic dye indocyanine green (ICG) concerns an interesting candidate given its history in the ophthalmologic field as a dye to stain the ILM during ILM peeling. Moreover, it is favored with photothermal properties operating in the near-infrared (NIR) range which is beneficial in an in vivo setting [23,24,25,26]. Triggered by this potential match, we recently revealed the strength of the photosensitizing capacity of free ICG as proof of principle was established to disrupt the ILM via ICG-mediated photoporation resulting in enhanced retinal drug delivery [27]. Although these results highlight the potential of this photodisruption concept, several aspects must be considered in view of clinical translation. Firstly, after IVT injection, ICG might be captured into the network of the vitreous cavity due to its affinity for collagen resulting in loss of a fraction of ICG that is able to reach the ILM. Thus, laser application can potentially affect the collagen network of the vitreous. Secondly, due to its molecular weight of 775 Da, ICG is able to cross the ILM and enter the retina implying that the VNB effects might not remain restricted to the ILM [28]. The current design of the treatment could, consequently, induce collateral damage at the level of retinal cells and/or vitreous. Therefore, to boost this promising concept to a further extent, we intended to load ICG into nanoparticles unable of crossing the ILM to strive for a more localized treatment and smoothen the migration through the vitreous.

Interestingly, owing to ICG’s profitable spectral properties, incorporation of ICG into NPs is a highly investigated topic; It is researched in context of light triggered drug release, imaging purposes, photodynamic and photothermal therapy for cancer treatment. As a result, an expanded set of NP designs are discussed in literature as ICG is compatible with different materials attributable to its amphiphilic nature—e.g., mesoporous silica [29], polymeric [30], lipid [31]- as well as protein-based [32] nanoparticles. As IVT injection of ICG NPs would be preferred, optimal mobility in the vitreous should be guaranteed which is dictated by the size and surface charge of NPs. In this regard, a size below 550 nm is required to be able to traverse through the collagen network while a negative to neutral charge should prevent binding to vitreous components [33,34]. On top of that, a second criterium implies that ILM penetration of ICG NPs must be excluded requiring a size above 100 nm [35,36]. Accordingly, to design the ideal NP for our approach the following criteria must be met: (1) a negative to neutral charge and a size below 550 nm to allow mobility through the vitreous, (2) a size above 100 nm to prevent ILM penetration as well as (3) a high ICG loading for efficient VNB generation.

**Figure 1:**
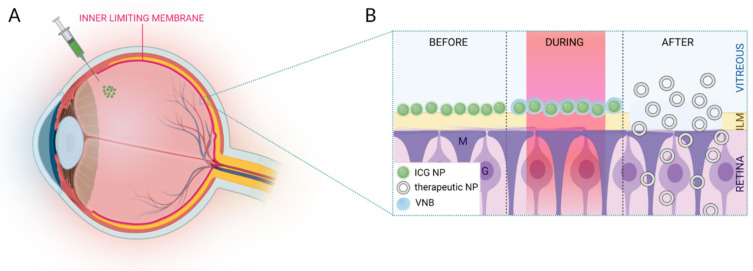
(**A**) Schematic overview of IVT injection of ICG NPs. (**B**) After migration through the vitreous, ICG NPs accumulate at the level of the ILM. Application of high intensity laser pulses results in VNB formation. Upon collapse of these VNBs, mechanical effects can disrupt the ILM paving the way for therapeutics to enter the retina. Image created with BioRender.

In this study, we explore two types of ICG NPs with biodegradable and biocompatible characteristics for photodisruption of the ILM (Figure 1). We incorporated ICG in different types of poly (lactic-co-glycolic acid) (PLGA) NPs and liposomes which were screened in terms of physicochemical and ICG incorporating characteristics. The VNB creating capacity was furthermore investigated by use of dark field microscopy in two different settings: in buffer and accumulated onto patient-derived human ILM. Since ICG liposomes emerged as the most encouraging photothermal NP, their VNB effects were finally evaluated in an ex vivo setting on bovine retinal explants.

## 2. Experimental Section

### 2.1. Materials

1,2-dipalmitoyl-sn-glycero-3-phosphocholine (DPPC), 1,2-distearoyl-sn-glycero-3-phosphocholine (DSPC), 1-stearoyl-2-hydroxy-sn-glycero-3-phosphocholine (Lyso PC), 1,2-distearoyl-sn-glycero-3 phosphoethanolamine (DSPE), 1,2-distearoyl-sn-glycero-3-phosphoethanolamine-N-[methoxy (polyethylene glycol)-2000] (DSPE-PEG), 1,2-dioleoyl-3-trimethylammonium-propane (DOTAP) and cholesterol were purchased from Avanti Polar Lipids, Inc. (Alabaster, AL, USA). Poly (vinyl alcohol) (PVA, M_w_ 13,000–23,000) 88–89% hydrolyzed, poly (D,L-lactide-co-glycolide) (PLGA, M_w_ 7000–17,000) 50:50, dimethylsulfoxide (DMSO) and acetonitrile were obtained from Sigma-Aldrich (St. Louis, MO, USA). Dichloromethane was purchased from Carl Roth (Karlsruhe, Germany). Paraformaldehyde (PFA) and methanol were purchased from Honeywell Research Chemicals (Charlotte, NC, USA).

### 2.2. Synthesis of ICG NPs

#### 2.2.1. ICG Liposomes

Three types of ICG liposomes composed of varying lipid components were synthesized via the thin-film hydration method based on previous reports in literature [36,37]. Lipids were dissolved in chloroform and mixed in different ratios (Table 1). The lipid mixture was placed in a rotary evaporation system to exclude the organic solvent by gradually reducing the pressure below 100 mbar at 40 °C. Next, the lipid film was hydrated with 500 µL of a water phase composed of ICG dissolved in HEPES (N-2-hydroxyethylpiperazine-N-2-ethane sulfonic acid) buffer (20 mM, pH 7.4). To solubilize the lipid film, this hydration step was performed in a water bath at 60 °C for 1 h under continuous rotation. Subsequently, the liposomes were downsized by applying a tip sonication cycle (3× 10 s, 10% amplitude (A), Branson digital sonifier, Danbury, CT, USA). After removal of free components by dialysis for 24 h at 4 °C (20 kDa, Float-A-Lyzer^®^ G2, Spectrum laboratories), the ICG liposomes were stored at 4 °C.

#### 2.2.2. PLGA ICG NPs

Based on the spontaneous emulsification solvent evaporation method, three types of PLGA ICG NPs were investigated according to previous described protocols with slight modifications [38,39,40]. A detailed overview of the solvents, volume ratios as well as ICG, PLGA and PVA concentrations for each type is displayed in Table 2. Briefly, varying amounts of PLGA and ICG were independently dissolved in an appropriate organic solvent and gently pooled together to obtain the organic phase. Next, the ICG/PLGA mixture was added dropwise to the aqueous phase composed of MilliQ water with varying concentrations of PVA as a stabilizer. For type 2 specifically, a proper emulsion was obtained by an extra tip sonication step (10 s, 10% A). Next, the organic phase was allowed to evaporate by continuous stirring for 4 h at room temperature. In order to collect the PLGA ICG NPs and eliminate free components, 3 washing steps with MilliQ water were performed by use of centrifugation (type 1:3 min at 4000× *g*; type 2:5 min at 7000× *g*; type 3:5 min at 10,000× *g*).

### 2.3. Characterization of ICG NPs

#### 2.3.1. Size and Zeta Potential

Dynamic light scattering (DLS, Zetasizer Nano ZS, Malvern instruments Co., Worcestershire, UK) was used in order to screen the hydrodynamic diameter as well as the zeta potential of ICG NPs. Prior to analysis, ICG NPs were diluted 1/100 in HEPES buffer for ICG liposomes or MilliQ water for PLGA ICG NPs. The size distribution was reported as the Z-average value.

#### 2.3.2. ICG Concentration and Encapsulation Efficiency

The ICG concentration was determined based on fluorescence measurements by use of a microplate reader (Victor^3^, PerkinElmer, Waltham, MA, USA). In order to measure the encapsulated amount of ICG, ICG NPs were dissolved in DMSO to release the ICG. Based on a standard curve (0.08–5 µg/mL) of free ICG in DMSO, we were able to measure the ICG concentration of the individual ICG NPs. The encapsulation efficiency (EE%) was furthermore calculated based on Equation (1).

(1) $$\mathrm{EE}\%=\frac{\mathrm{Initial}\;\mathrm{mass}\;\mathrm{ICG}}{\mathrm{mass}\;\mathrm{ICG}\;\mathrm{in}\;\mathrm{formulation}}\times 100\;$$

### 2.4. VNB Generation ICG NPs in Buffer and on Isolated Patient-Derived Human ILM

To determine whether ICG NPs are suitable photothermal entities to induce ILM photodisruption, VNB generation was evaluated in two different settings: ICG NPs in buffer as well as ICG NPs accumulated onto the ILM. Depending on the type of particle, a proper dilution in MilliQ or HEPES buffer was made to be able to visualize potential VNBs. In case of studying the effects on the ILM, patient-derived human ILM isolated during ILM peeling was obtained from Ghent University Hospital. Protocols were approved by the Ethical Committee of Ghent University Hospital (dossier number: BC-10642). After an incubation period of 15 min for the NPs to sediment and/or attach to the ILM, VNBs were detected via dark field microscopy. Since the home built 800 nm picosecond set-up is not connected to a dark field microscope, another laser set-up was used to detect VNB formation. With this set-up, laser pulses of 7 ns were applied tuned to a wavelength of 561 or 647 nm (Opolette HE 355 LD, OPOTEK Inc., Carlsbad, CA, USA). The laser pulse energy was monitored by an energymeter (J-25MB-HE&LE, Energy Max-USB/RS sensors, Coherent, Santa Clara, CA, USA) synchronized with the pulsed laser. Images and/or movies were recorded via MicroManager and Free Cam software, respectively.

### 2.5. Isolation of Bovine Retinal Explants

The entire procedure of explant isolation, laser treatment, cryopreservation, sectioning and immunostaining was performed as described earlier [27]. Bovine eyes were obtained from a local slaughterhouse and transported in cold CO_2_ independent medium (Gibco^®^, Paisly, UK). The excess muscle and glandular tissue were removed in order to allow for a smooth dissection. After submerging the eye in 20% ethanol, the sclera was punctured 10 mm below the limbus with a 21G needle to create an entrypoint to bisect the eye with curved scissors followed by separation of the anterior segment. After gently removing the vitreous, the posterior eyecup was filled with CO_2_ independent medium. Three relaxing cuts were made to flatten the entire structure to be able to isolate retinal explants by use of an 11 mm trephine blade (Beaver-Visitec International, Waltham, MA, USA). After carefully removing the surrounding retina, the explants were isolated by pipetting CO_2_ independent medium below the explants.

### 2.6. Laser Treatment of Bovine Retinal Explants

Bovine retinal explants were transferred to a 35 mm glass bottom dish (Nunc™, Thermo Fisher Scientific, Waltham, MA, USA) making sure the ILM was positioned upwards. The dish was placed in the laser set-up and 20 µL of ICG NPs was added on top of the explants. Next, the entire explant was scanned with 2 picosecond laser pulses of 800 nm at a frequency of 1 kHz. For this purpose, a home built set-up was used powered by a Ti:Sapphire regenerative amplifier (Spitfire-Ace PM1K, Spectra-Physics, Milpitas, CA, USA) seeded by a Ti:Sapphire solid state laser (Mai Tai HP, Spectra-Physics, Milpitas, CA, USA) and pumped by a diode Nd:YLF laser (Ascend 40, Spectra-Physics, Milpitas, CA, USA). The total scanning time of one retinal explant was approximately 6 min. After laser treatment, the fixation and cryopreservation process was immediately initiated.

### 2.7. Cryopreservation, Sectioning and Immunostaining

After completing the laser treatment, bovine retinal explants were fixed with 4% PFA for 2 h at 4 °C. After removal of the PFA, cryopreservation was performed which includes three steps. Sequentially, retinal explants were incubated with 30% sucrose overnight (4 °C), 1:1 30% sucrose/O.C.T (Tissue Tek^®^, Sakura Finetek, Antwerp, Belgium ) for 3 h (4 °C) and O.C.T for 3 h (25 °C). Next, the explants were transferred to cryomolds embedded in fresh O.C.T followed by snap freezing in cooled isopentane and stored at −20 °C. Using a cryostat (Leica), 10 µm cryosections were cut followed by mounting onto SuperFrost^®^ Plus microscopy slides (Thermo Fisher Scientific, Waltham, MA, USA). In order to obtain an overview of the entire retina, 6 different locations at a distance of 1 mm from each other were investigated per sample.

The ILM was visualized by use of an indirect immunostaining method with collagen IV antibodies, a main constituent of the ILM. As a first step, the sections were washed 10 min with phosphate buffered saline (PBS) at room temperature. Next, the tissue was permeabilized by submerging the sections in 0.1% Triton for 5 min followed by another washing step with PBS for 10 min. After a blocking step with 5% goat serum for 1 h at room temperature, the sections were incubated overnight with a 1:200 dilution of rabbit anti-collagen IV antibody (Ab6586, Abcam, Cambridge, UK) at 4 °C. The primary antibody was removed by a washing step with PBS for 10 min. Subsequently, the sections were incubated with a 1:500 dilution of Hoechst as well as secondary anti-rabbit AlexaFluor 568 antibody (Invitrogen). A last washing step in PBS was executed to finally mount the slides with 1:1 PBS/glycerol solution. Retinal cryosections were imaged via confocal microscopy (Nikon A1R) using a 60x water objective (SR plan apo IR 60X WI). Further processing of the images was achieved with Fiji software.

## 3. Results

### 3.1. Synthesis of ICG Nanoparticles with Suitable Physicochemical Characteristics

In the search for a suitable nanosized-photosensitizer for localized ILM photodisruption, two main classes of ICG NPs with biodegradable and biocompatible features were evaluated: ICG liposomes and PLGA ICG NPs. By varying several parameters during synthesis, three individual sub-types were investigated for each class (Table 1 and Table 2).

**Figure 2:**
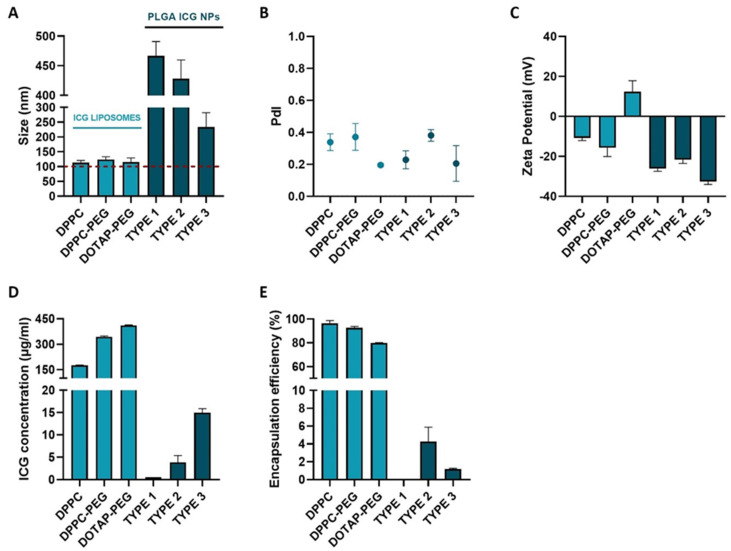
Physicochemical and ICG incorporating characterization of ICG liposomes (DPPC, DPPC-PEG and DOTAP-PEG) and PLGA ICG NPs (types 1–3). (**A**) Hydrodynamic diameter. (**B**) PdI. (**C**) Zeta Potential. (**D**) Total ICG concentration. (**E**) Encapsulation efficiency of ICG.

As proper physicochemical characteristics are of utmost significance to reach the ILM after IVT injection, the different ICG NP formulations were screened in terms of size and charge by measuring the hydrodynamic diameter and zeta potential, respectively. Based on Figure 2A, it can be noted that all the ICG NPs exceed the lower size limit criterium of 100 nm to be able to accumulate at the level of the ILM. While the sizes of all ICG liposomes are consistently leaning towards the lower limit independent of the lipid composition, with values ranging between 113 ± 8 nm and 124 ± 9 nm, PLGA ICG NPs are clearly found in a higher size-order. In the latter class, amending the organic solvents, PLGA and PVA concentration during synthesis gave rise to relevant differences. Type 1 and 2 resulted in the largest particles with values of 467 ± 24 nm and 428 ± 32 nm, respectively, in vicinity of the upper size limit of 550 nm. On the contrary, type 3 is characterized by a significantly lower size of 233 ± 48 nm.

In addition to size, surface charge is considered as another key parameter dictating the fate of NPs after IVT injection. According to literature, it is known that a negative to neutral charge is favorable to permit sufficient mobility in the vitreous [41]. When analyzing the zeta potential, Figure 2C indicates that all ICG NPs meet this requirement–except for the DOTAP-PEG liposomes. All PLGA ICG NPs were found to be highly negatively charged (<−20 mV) independent of changed parameters during synthesis, while the charge of ICG liposomes is lipid dependent. Evidently, incorporating neutral lipids such as DPPC and DSPC should result in neutral liposomes. However, due to incorporation of negatively charged ICG in the lipid layer this value shifted to slightly negative values (>−20 mV). When changing to a positively charged lipid system, the overall surface charge of the DOTAP-PEG liposomes was found to be slightly positive.

As sufficient ICG encapsulation could possibly be a prelude to identify the most successful particle in terms of VNB creating capacity, the ICG concentration as well as the encapsulation efficiency (EE%) of ICG NPs were evaluated (Figure 2D,E). Remarkably, ICG liposomes indistinctly outclass PLGA ICG NPs in terms of both parameters. Type 1 PLGA ICG NPs embodies the least impressive candidate as both values of ICG concentration and EE% are almost negligible. Although types 1 and 2 perform slightly better, the highest ICG concentration in this class of NPs does not exceed 15 µg/mL and encapsulation efficiencies remain below 10% indicating that a large fraction of ICG is lost during synthesis. Interestingly, ICG liposomes were able to ascend ICG concentrations to 180 µg/mL for DPPC liposomes and even 343 µg/mL for DPPC-PEG liposomes combined with efficient encapsulation with values above 90%. Although for DOTAP-PEG liposomes higher concentrations up to 411 µg/mL were obtained, it is of note that this is probably due to the increase of the lipid concentration. Hence, the number of liposomes is probably higher compared to the DPPC-(PEG) liposomes. Moreover, the encapsulation for this type of liposome was the lowest within this class with a value of 79.8%.

### 3.2. ICG Liposomes Are Manifested as the Most Promising Photosensitizer

**Figure 3:**
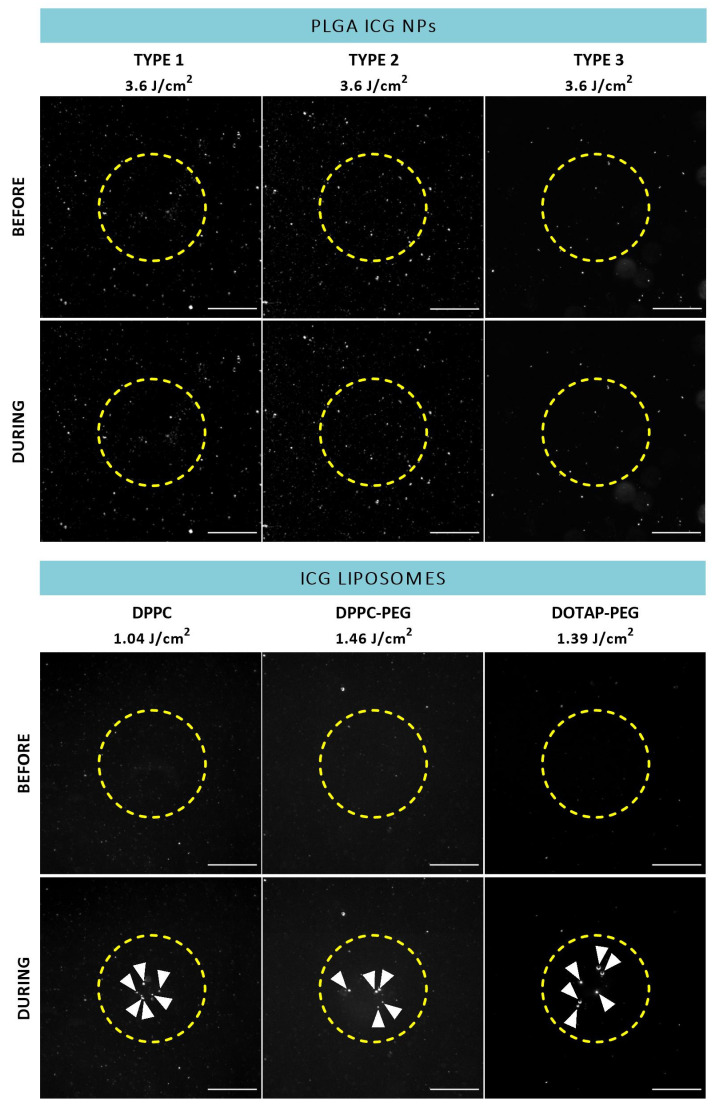
Dark field microscopy images of ICG NPs in buffer before and during application of a 7 ns laser pulse (ICG liposomes: 647 nm, ~1.3 J/cm^2^, PLGA ICG NPs: 561 nm, ~3.6 J/cm^2^). White arrowheads indicate the presence of VNBs. Yellow dotted line represents the size of the laser beam. Scale bar = 75 µm.

To reveal whether ICG NPs display intrinsic VNB creating capacities, dark field microscopy was performed before and during application of a 7 ns laser pulse to detect the presence of VNBs for all formulations in buffer solution (Figure 3). In this regard, the suspected trends founded by the ICG incorporating characteristics are largely confirmed. Indeed, PLGA ICG NPs are not able to generate VNBs despite application of powerful 3.6 J/cm^2^ pulses of 561 nm indicating ICG concentrations lower than 15 µg/mL do not suffice for the rapid temperature increase to generate VNBs. On the contrary, the three types of liposomes emerge as efficient mediators of photodisruption since bubble formation was observed (Figure 3, white arrowheads). In order to acquire an impression of differences in efficiency between the individual types of ICG liposomes, the lowest laser fluences able to induce apparent VNB formation were determined. Therefore, the wavelength was tuned to 647 nm to approximate ICG’s absorption maximum of 800 nm as close as possible. However, no appreciable difference is observed as the values remain between 1.04 and 1.46 J/cm^2^ for all liposomes. Lastly, another important remark to make is that, given the size of the liposomes, the observed generated bubbles are possibly not originating from individual liposomes but rather provoked by aggregation of NPs.

**Figure 4:**
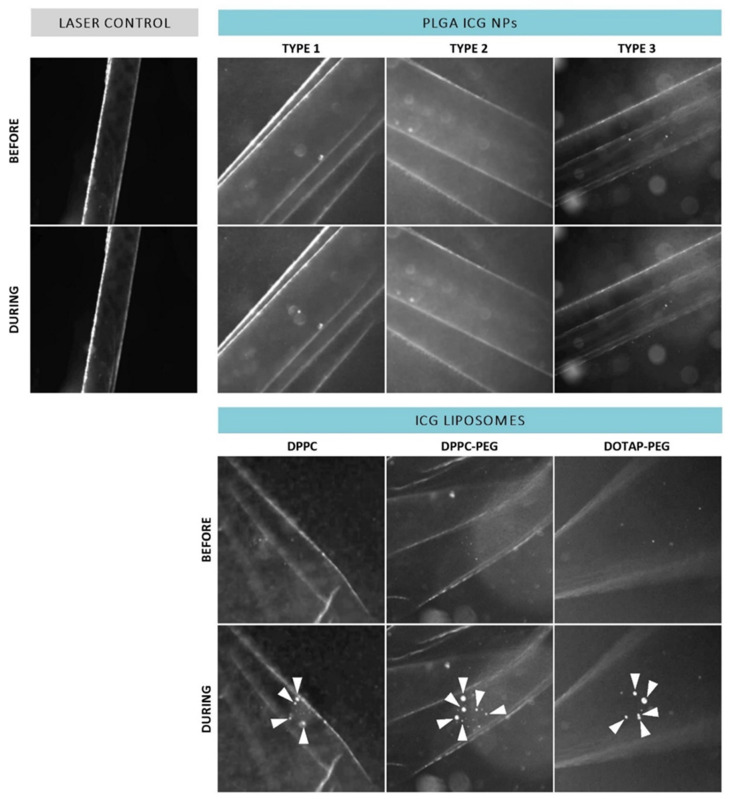
Dark field microscopy images of patient-derived isolated human ILM incubated with ICG NPs before and during application of a laser pulse (561 nm, ~3.6 J/cm^2^). White arrowheads indicate presence of VNBs.

Since the actual target of ICG NP-mediated photodisruption is in a biological environment, the interaction between ICG NPs and the ILM could possibly change the observed effects. Therefore, the same process was repeated after incubation with patient-derived isolated human ILM as displayed in Figure 4. Although accumulation of PLGA ICG NPs onto the ILM could possibly boost VNB generation, there was still a lack of VNBs for each type. On the other hand, the three different types of ICG liposomes were still able to form VNBs at the human ILM. However, when revolving around effects on the ILM itself after VNB formation, no persuasive impact such as pore formation is observed for any type of ICG liposome as the human ILM seems to remain intact.

**Figure 5:**
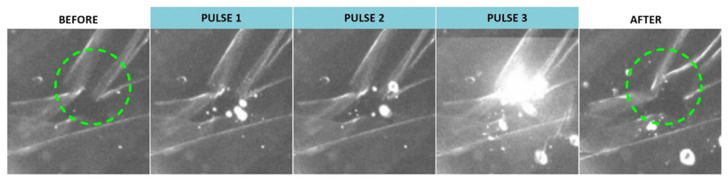
Dark field microscopy images of patient-derived ILM incubated with DPPC-PEG liposomes before, during and after application of three individual laser pulses (561 nm, ~3.6 J/cm^2^). Green dotted circle indicates structural changes of the ILM.

Although application of a single pulse resulted in creation of VNBs but did not elicit noticeable effects at the level of the ILM, we investigated whether this could be improved by use of repeated pulses. Remarkably, we did observe some effects when multiple pulses were applied as highlighted in Figure 5. Application of three individual pulses resulted in some structural changes as indicated by the green dotted circle. However, this feature was only obtained for DPPC-PEG liposomes probably due to the fact that the ICG incorporation per individual liposome is the highest compared to the other liposomes based on the ICG:lipid ratio.

### 3.3. ILM Integrity Is Slightly Affected after Laser Treatment with ICG Liposomes

**Figure 6:**
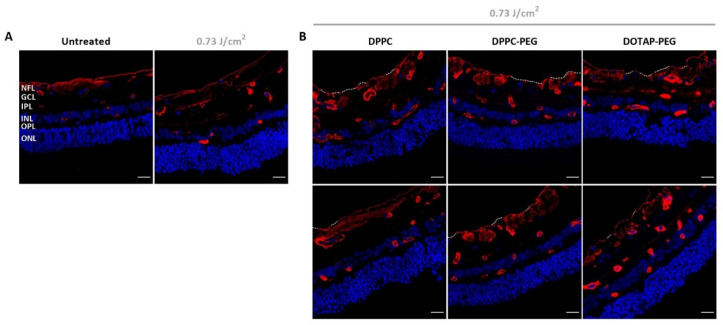
Visualization of retinal cryosections via confocal microscopy. (**A**) Control samples: untreated and laser treated. (**B**) Treated samples: application of ICG liposomes on top of bovine retinal explants followed by laser treatment with 800 nm pulsed laser light. Blue: Hoechst staining to visualize nuclei. Red: immunostaining for Collagen IV to show the ILM and blood vessels. White dotted line indicates complete absence of the ILM. Scale bar = 20 µm.

To further investigate the possible impact of our treatment on ILM integrity from a different perspective, the approach was tested on bovine retinal explants. To this end, a picosecond setup was used tunable to a wavelength of 800 nm matching the absorption maximum of ICG. As based on the preceding experiments ICG liposomes surfaced as the most promising photothermal agent, the next set of experiments was continued with this class of NPs. After laser treatment, bovine explants were further processed into retinal cryosections stained with collagen IV antibodies, a main constituent of the ILM. As depicted in Figure 6A, an intact ILM is observed in untreated samples. Similar observations are made when explants underwent laser treatment without the presence of a photosensitizer implying that laser only is insufficient to affect the ILM. Interestingly, we did observe subtle effects when laser treatment was mediated with ICG liposomes as clarified in Figure 6B (two representative images). For all ICG liposomes, regions can be found where a large portion of the ILM is fully ablated (top row, white dotted lines). However, locations where the ILM remains mainly intact (bottom row, few dotted lines) are also present. It is of note that in the latter case it appears that the ILM occurs less bright compared to control samples. Interestingly, this observation can denote that an “ILM thinning” effect, where part of the ILM is removed without fully ablating it, might be provoked by our treatment. For each tested NP, both ILM ablation and thinning are occurring in approximately equal proportion concluding that no specific ICG liposome is surpassing its competitors. In general, the structural organization of the retina is retained in all cases.

## 4. Discussion

Visual perception is enabled by an intricate process which involves entry of light in the eye and its translation into an image at the level of the brain. To bring this to a successful conclusion, the neurosensory retina vouches for the key task to translate light into an electric message. Unfortunately, several acquired and inherited disorders find their origin at the level of the retina. Yet, the evolving expertise in the field of nucleic acid based- and stem cell therapy opens increasing perspectives to develop promising therapeutics to combat retinal diseases. While PRs and RPE cells located in the outer retina have a well-known history as target cells for gene augmentation strategies, other interesting modes of action such as neuroprotection, optogenetics and reprogramming require inner retinal targeting. To reach these cells, IVT injection is the most attractive delivery route from a targeting as well as a safety perspective [8]. Large therapeutics are, unfortunately, in many cases blocked at the level of the ILM while heading for the retina. Excitingly, our research group recently revealed the power of ICG-mediated photodisruption to bypass this barrier in a controlled and tunable manner [27]. Although this concept has proven its worth to enhance retinal drug delivery, opportunities to further boost or fine-tune the technique more in depth are still in place. A potential hurdle in view of clinical translation is that, due to affinity of ICG for collagen in the vitreous and its ability to diffuse into the retina, ICG might not efficiently reach or remain strictly localized at the ILM which may lower the potency of the technique or induce collateral damage, respectively. To eliminate these potential concerns, we explore loading of ICG into NPs unable of crossing the ILM to efficiently guide ICG in the direction of the ILM aiming for a localized treatment.

In order to pursuit localized disruption effects at the level of the ILM, optimization of a suitable ICG-nanocarrier is the first pivotal step. To be able to traverse through the mesh network of the vitreous after IVT injection a size below 550 nm and negative to neutral charge are a must. On top of that, ILM passage should be avoided by targeting a size above 100 nm. Two main classes of biodegradable and biocompatible NPs eligible to meet these requirements were scrutinized: ICG liposomes and PLGA ICG NPs. For each individual class, three independent sub-types were evaluated to find the ideal match for our approach. Interestingly, all screened formulations were characterized by a size within our effective window of 100–550 nm. ICG liposomes were all featured by a size narrowly exceeding the lower limit of 100 nm, independent of the lipid composition, ICG and lipid concentration. On the other hand, PLGA ICG NPs clearly yielded larger particles yet remaining below the 550 nm upper limit. Since the PLGA ICG NPs were all synthesized based on emulsification, the NP size is mainly dependent on the stability of the emulsion droplets governed by several formulation parameters such as the type of solvents, polymer and stabilizer concentration [42,43,44]. By varying several of these parameters, the size could be tuned to large sizes for type 1 and 2 (>400 nm) while type 3 resulted in significantly smaller NPs (~233 nm) which is in accordance with the differences observed when comparing the respective sizes obtained by other research groups [38,39,40]. In terms of kinetic mobility, it is evident that given their larger size PLGA ICG NPs type 1 and 2 will migrate at a slower pace through the vitreous which potentially lowers the odds to effectively reach the ILM compared to the other ICG NPs. While diffusion might be retarded to some extent in consequence of size, different reports in literature state the importance of surface charge of NPs as the main decisive factor for diffusion behavior [33,45,46]. Indeed, as the vitreous is a negatively charged network, positively charged nanoparticles might be immobilized to a large extent due to electrostatic interaction while negative and neutral NPs are in general not restricted. Interestingly, all formulations were marked by a slightly negative to negative charge, except for the DOTAP-PEG liposomes which are slightly positively charged. Yet, in the latter case, an extra coating step with negatively charged components, e.g., hyaluronic acid, might facilitate vitreal migration [47]. Since, as evidenced by Martens et al., PEGylation of NPs might improve the diffusion rate even more, DPPC-PEG liposomes might be most preferred in terms of mobility in the vitreous.

Although ICG NPs might be equipped with the desired physicochemical characteristics, sufficient incorporation of ICG into the NP is possibly a critical factor to ensure ILM destructing capacities. Therefore, ICG concentration and encapsulation efficiency were evaluated as indicator to predict whether ICG NPs can operate in the VNB mode. ICG liposomes were found to be discernibly more skilled to incorporate higher ICG amounts, ranging between 180 and 411 µg/mL, compared to PLGA ICG NPs which are not able to exceed values of 15 µg/mL. For the latter class attempts to increase ICG incorporation by varying synthesis parameters were unsuccessful. As the synthesis of PLGA ICG NPs was performed based on emulsification, it is likely that ICG does not remain confined to the organic phase based on its amphiphilic nature resulting in loss of a large fraction of ICG. Additionally, as both PLGA and ICG are negatively charged, electrostatic repulsion might hamper proper incorporation. For ICG liposomes, on the contrary, ICG’s amphiphilic properties are profitable as it is reported that ICG is able to interact with phospholipids as well as liposomes [25]. While it is estimated that ICG is inserted in the lipid bilayer [48], Lajunen et al. revealed via molecular dynamics simulation that ICG is able to interact with hydrophilic PEG chains present on the surface of PEGylated liposomes [31]. The fact that we were able to incorporate a higher ICG concentration into PEGylated DPPC liposomes compared to their PEG-lacking counterparts accompanied by a more negative zeta potential is underpinned by this hypothesis. In an attempt to increase ICG concentration, positively DOTAP-PEG liposomes were investigated to explore the contribution of electrostatic interaction. Although the group of Miranda et al. claimed almost full complexation of ICG, we observed a significantly lower EE value of 79.8%. Considering the difference between the loading method, it is plausible that our passive loading could be less efficient. It should be taken into account that although the absolute ICG concentration of this type is the highest, the lipid concentration of this type of liposome was elevated whereby the ICG loading per individual particle is potentially lower. In this regard, the highest value is probably achieved with the DPPC-PEG formulation.

When turning to photothermal effects, two “modes” can be distinguished: direct heating mode and VNB mode [15,16]. While the direct heating mode is generally observed with continuous wave (CW) irradiation or low intensity pulsed laser light inducing thermal effects, high intensity laser pulses can provoke the creation of nanoscopic bubbles resulting in mechanical phenomena. Although the state-of-the art photothermal agents are often plasmonic NPs such as AuNPs favored with substantial absorbing capacities based on their localized surface plasmon resonance (LSPR) [14,15,16], these characteristics do not apply for ICG NPs. However, owing to ICG’s absorbing capacities in the NIR range, ICG NPs are widely investigated as photothermal NPs in the field of photothermal therapy to induce hyperthermia after CW irradiation to destroy tumor cells. These findings have spurred to investigate how ICG NPs behave after irradiation with high intensity laser pulses. In buffer solution, PLGA ICG NPs were not found to trigger VNB generation probably due to the low concentrations of ICG encapsulated into this type of NP (between 0.5 and 15 µg/mL). As a result, this class of particles is possibly only able to operate in the direct heating mode. In this regard, Della Pelle et al. observed a modest temperature rise of 7.5 °C when irradiating a 5 µg/mL ICG solution for 5 min with a CW laser (3 W/cm^2^, 808 nm) while further ascending to 50 µg/mL was accompanied by an elevated temperature rise of 50.7 °C [49]. This indicates that, although the actual temperature increase might be higher due to the use of high intensity laser pulses in our case, this is not sufficient to induce the rapid temperature increase needed for VNB generation. Although we believe that operating in the heating mode might be able to permeabilize the ILM to some extent as thermal denaturation of ILM proteins might be provoked, reaching the VNB mode is an advantage both in terms of efficacy and safety. Sauvage et al. demonstrated that less laser pulses are required to fully destroy vitreous floaters when the bubble mode is achieved. Most importantly, since Haritoglou et al. observed severe disorganization of the human inner retina after application of ICG followed by halogen illumination, the close proximity of the retinal cells makes us strive to reach the VNB mode to tune the approach as safe as possible [50]. Due to the insulating nature of gas combined with the very short bubble lifetime of VNBs, dissipation of heat to the surrounding tissue is negligible which should temper retinal toxicity. Intriguingly, this objective was achieved when irradiating ICG liposomes in buffer with 7 ns laser pulses. Based on the feature of ICG liposomes to incorporate higher amounts of ICG, higher increases in temperature are possibly obtained able to exceed the critical temperature of the buffer to induce evaporation. Interestingly, these values are in agreement with the ICG concentrations found to be effective in inducing VNB formation after accumulation of free ICG onto the ILM (0.1 mg/mL) and vitreous floaters (0.5 mg/mL) [19,27]. Given the size of the liposomes and number of bubbles, however, it is possible that we are not able to detect individual VNBs as it is more difficult to form VNBs with smaller NPs [22], but observing VNBs resulting from aggregated liposomes.

In a next step, to investigate possible changes in VNB-behavior in a biological setting and to detect structural damage at the level of the ILM, the same set of experiments was performed in presence of patient-derived human ILM. However, the same trends were sustained. While PLGA ICG NPs were still lacking the capacity to induce VNB formation, ICG liposomes preserved their assets to do so. Despite VNB generation for all ICG liposomes, no discernible effects at the level of the ILM were elicited such as the creation of holes as earlier observed for free ICG [27]. Since free ICG molecules, renowned for their high affinity for the human ILM, are able to cluster in the ILM-network, ICG NPs are rather “in touch” with the membrane implying that larger VNB formation should be obtained to provoke more pronounced effects. Yet, it is probable that more subtle effects, e.g., permeabilization or structural disorganization of the protein network of the membrane, are not retraceable via dark field microscopy. Remarkably, DPPC-PEG liposomes were able to disrupt the ILM to some extent after repetition of 3 individual laser pulses which was not the case for the other types of liposomes likely attributable to the highest ICG:lipid ratio of this liposome. While the efficacy of the approach might be upgraded by use of multiple pulses, a downgrade in terms of toxicity cannot be excluded.

Since ICG liposomes outperformed their polymeric PLGA NP counterparts at each stage, only ICG liposomes were included to evaluate ICG NP-mediated photodisruption on bovine retinal explants. While zones with absent parts of ILM were observed, areas with intact ILM were retrieved in almost equal amounts. Based on empirical evaluation, we suggest that the intact ILM might be thinned to some extent. Since the same trend was observed for all liposomes, none of them emerges as the most efficient photosensitizer which is expected based on the insignificant difference in the lowest laser fluence able to elicit VNBs. The fact that we were only able to observe mild effects, lacking a clear trend, might be explained by several hypotheses. Firstly, since leakage of ICG from the liposomes was not determined, we can not exclude the contribution of free ICG to the observed results. Secondly, as previously mentioned, we suggest that VNBs are rather generated due to aggregation of individual NPs. The difficult to control nature of this process might explain the irregularity of the observed effects. Lastly, another important parameter determining the probability of success is the affinity of the ICG NPs for the ILM. Since the ILM itself is considered to be negatively charged, electric repulsion might limit the affinity of the NPs for the ILM. However, since the ILM is a complex matrix composed of different extracellular proteins [9] where other mechanisms such as hydrophobic interactions may come into play, it is difficult to predict which physicochemical properties are most suitable for optimal interaction with the ILM. Moreover, steric hindrance due to PEGylation might further interfere with the affinity. Seeing that mild effects were observed on bovine retinal explants while almost no damage is provoked on isolated human ILM, we expect that the mild effects are not powerful enough to permeabilize the significantly thicker human ILM. In conclusion, the current approach with ICG liposomes is not able to disrupt the ILM in a consistent and reproducible manner. However, since it is probable that we still miss out subtle effects undetectable via dark field microscopy or imaging of cryosections, no clear statements can be made whether or not the observed effects are adequate to boost retinal drug delivery which should be investigated more into depth with advanced microscopy techniques or evaluation of entrance of model drugs.

An important point to consider is that only one type of synthesis was evaluated for both ICG liposomes and PLGA ICG NPs as the goal was to screen different types of NPs. Nevertheless, we are aware that other types of synthesis are attractive in terms of upscaling, avoiding the use of organic solvents and surfactants, controlling the size or might improve the ICG encapsulation. Although increase of ICG concentration might boost VNB generation even more, our observations suggest that the major obstacle is the interaction between the ILM and the NPs. Active targeting strategies might solve this issue, however, attachment of antibodies to the liposomes is possibly challenging and this extra step can complicate market translation. On top of that, switching to other types of ICG NPs can be another perspective to intrinsically improve the affinity of ICG NPs for the ILM. While mesoporous silica NPs are also widely investigated to incorporate ICG, other types of NPs matching the biological environment of the ILM, e.g., albumin- or hyaluronic acid based NPs, or more neutrally charged ones could be promising. Hence, the possible influence of the nature and charge of ICG NPs on the interaction with the complex protein matrix of the ILM is an interesting topic which should be investigated more into depth.

## 5. Conclusions

The main objective of this study was to screen the potential of different types of ICG NPs as photothermal entities to boost the ILM photodisruption concept, previously established by our research group, in terms of restricting the treatment to the ILM only. In this way, collateral damage to other structures could be bypassed and toxicity concerns tempered. We were able to successfully synthesize a set of PLGA ICG NPs and ICG liposomes characterized by suitable physicochemical properties that qualify for our approach. ICG liposomes towered above PLGA ICG NPs in terms of ICG incorporating features which was shown to determine their potential to induce VNBs. Generation of VNBs was only observed for ICG liposomes both in buffer and after accumulation onto the human ILM. Nevertheless, laser treatment on bovine retinal explants mediated by ICG liposomes merely resulted in limited effects including small areas of ablated ILM alternated by zones of mainly intact but possibly thinned ILM. Whether or not these effects are satisfactory to enhance retinal drug delivery is the next question that should be answered. However, based on our results we hypothesize that the major obstacle involves the interaction between the ICG NPs and the complex ILM matrix, an important subject which demands further investigation. In addition to active targeting strategies of ICG liposomes to enhance this interaction, exploring other NP types with different building blocks and/or more neutral charges might gain more insight into NP-ILM interaction and hence improve the concept to a further extent. 

## Figures and Tables

**Table 1:** Composition, molar ratios and starting concentrations of ICG liposomes.

ICG LIPOSOMES				
Type	Composition	Molar Ratio	ICG Concentration (mg/mL)	Total Lipid Concentration (mg/mL)
DPPC	DPPC/DSPC/Lyso PC/DSPE	75/15/10/4	0.22	7.2
DPPC-PEG	DPPC/DSPC/Lyso PC/DSPE-PEG	75/15/10/4	0.37	7.2
DOTAP-PEG	CHOL/DOTAP/DSPE-PEG	10/9/1	0.53	25

**Table 2:** Solvents, starting concentrations and volume ratios of PLGA ICG NPs. (I: organic solvent ICG, P: organic solvent PLGA, O: total organic phase, W: total aqueous phase).

PLGA ICG NPs							
Type	Concentration ICG (mg/mL)	ICG Solvent	Concentration PLGA (mg/mL)	Solvent PLGA	PVA	Volume Ratio I/P	Volume Ratio O/W
1	10	Methanol	50	Acetonitrile	4%	1:1	1:4
2	1	Methanol/DCM	30	Methanol/DCM	0.25%	3:7	1:10
3	10	DMSO	10	Acetonitrile	-	1:10	1:5

## Data Availability

The data presented in this study are available in this article: Photodisruption of the Inner Limiting Membrane: Exploring ICG Loaded Nanoparticles as Photosensitizers.
